# Supplementary material for: Clinical-Grade Patches as a Medium for Enrichment of Sweat-Extracellular Vesicles and Facilitating Their Metabolic Analysis
Source: Int J Mol Sci. 2023 Apr 19;24(8):7507. doi: 10.3390/ijms24087507 (PMC10139190; doi:10.3390/ijms24087507)
Supplement: Supplementary file 1 [file ijms-24-07507-s001.zip › ijms-2248755-supplementary1.pdf]

Figure S1

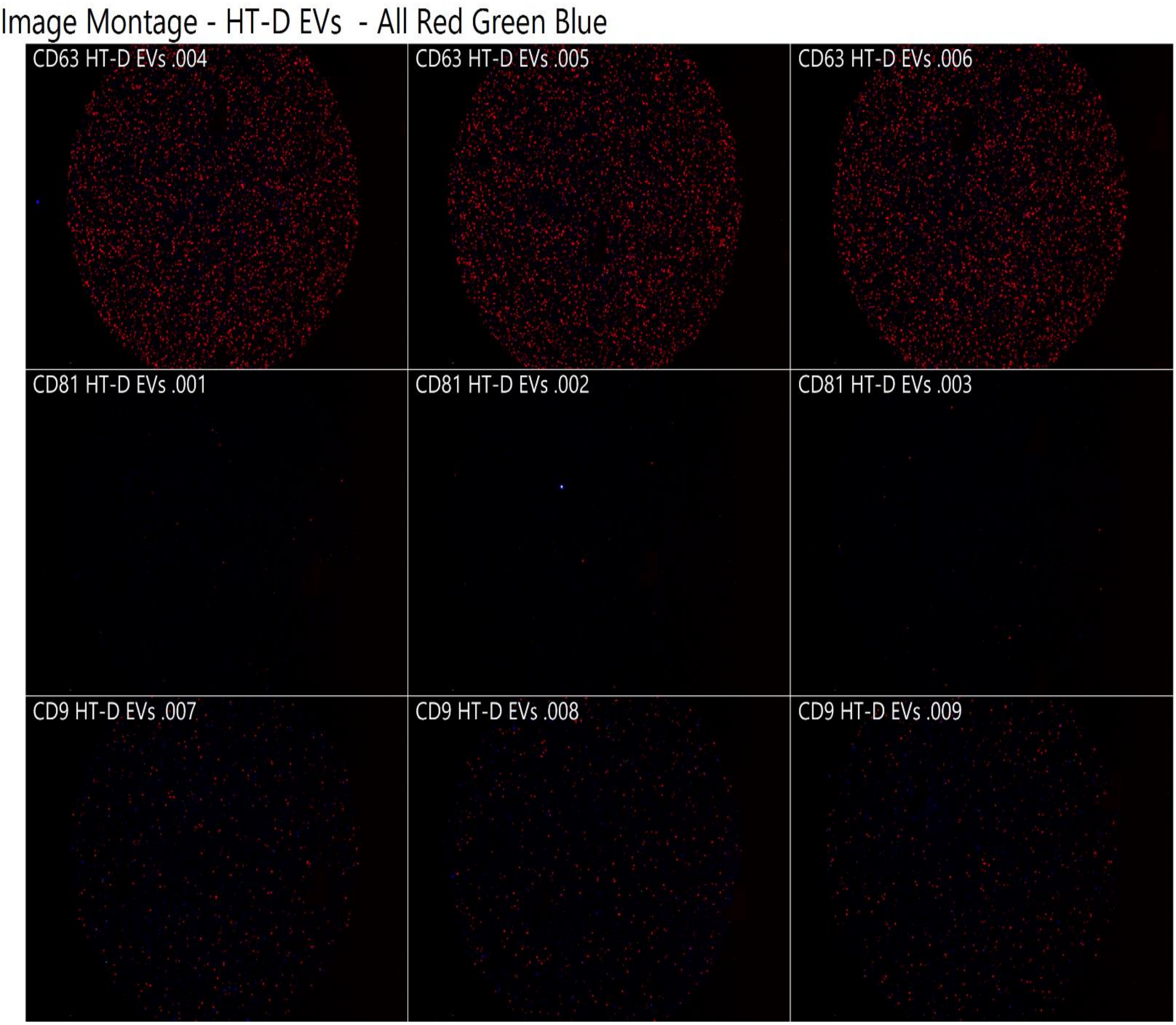
Image Montage - HT-D EVs - All Red Green Blue
CD63 HT-D EVs .004
CD63 HT-D EVs .005
CD63 HT-D EVs .006
CD81 HT-D EVs .001
CD81 HT-D EVs .002
CD81 HT-D EVs .003
CD9 HT-D EVs .007
CD9 HT-D EVs .008
CD9 HT-D EVs .009

Figure S2

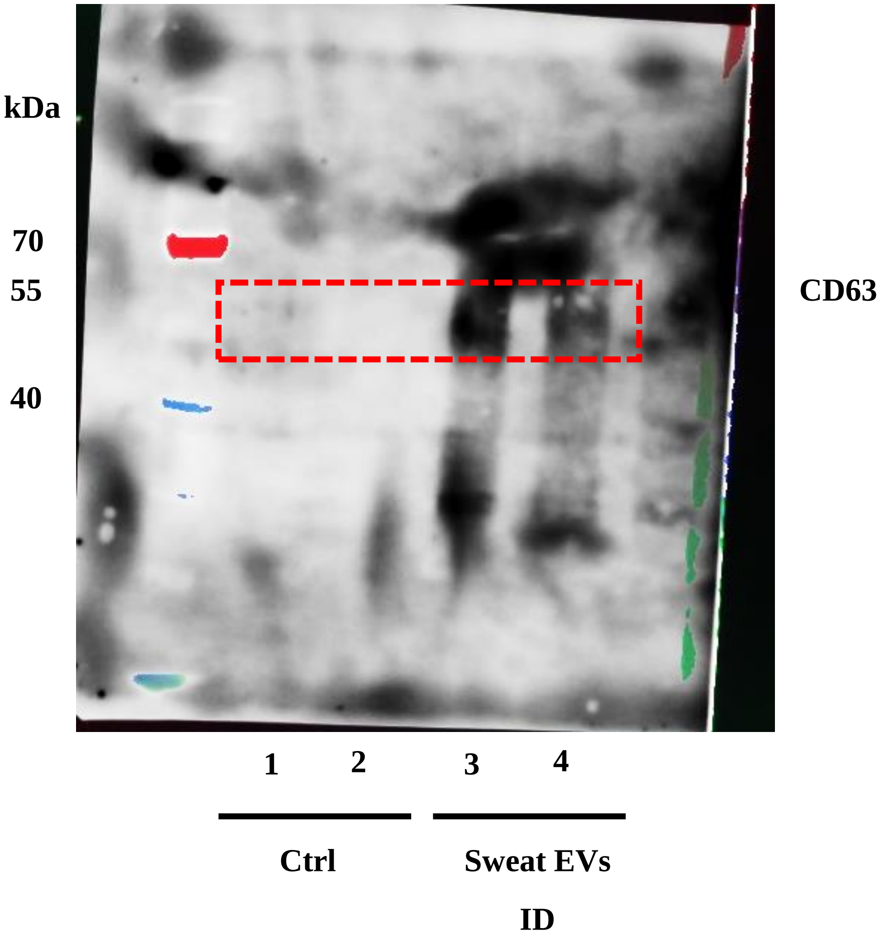

Table S1

	Samples											Negative control	
	**1**	**2**	**3**	**4**	**5**	**6**	**7**	**8**	**9**	**10**	**11**	1	2
**Pyroglutamate**	39054544	48165907	25556444	49849603	37140711	102096849	88218367	39399666	69990373	37224055	49808475	31507280	20522501
**Aspartate**	3115443	0	647124	1535215	3999005	5127384	2356554	638487	4477041	2388006	2259549	258249	749945
**Glycine**	3170680	3576517	1931887	3358687	31860404	5405309	15615591	3918535	123081860	3291529	3727417	1972018	4331971
**Alanine**	7212292	11888144	6090053	8243319	5963519	16263923	8831818	7638691	11931400	6163053	8550931	6953538	3288891
**Arginine**	7886835	11997376	5576911	7486309	4242414	12226811	7137445	3920171	5631930	3987141	3522258	5765546	2259310
**Asparagine**	681016	765714	475458	691295	706331	838793	692567	593186	961256	649512	668921	563041	365651
**Leucine**	6615509	14532803	5343495	6632030	4649717	21420101	6086452	5190486	11999143	5133818	7894918	5913102	3778215
**Glutamate**	4638434	3381467	2688340	3017000	4282510	5881854	3045374	2179740	5344199	2765681	2768268	1984387	1504963
**Glutamine**	4522324	5933238	3249098	3613586	4023261	5382305	2947484	2556130	4139030	3111610	3691629	1955429	1841153
**Linoleate**	5882792	9843666	5580933	4709378	6664589	10862756	6817957	8842856	6494111	4519039	6311114	7128317	6810048
**Isoleucine**	0	8089342	6563280	5551454	0	10803000	5523122	4750518	9465768	4565030	6180327	3303933	2398208
**Lactate**	284559438	272845698	178537347	173015930	302936960	255858763	525739242	348060464	189803171	134628770	153627469	124826856	140342051
**Lysine**	1631125	2110834	1006482	1399769	1359508	1255208	1154859	888615	806093	811886	1166344	1471838	978019
**Methionine**	339733	1954141	246382	394913	493638	2088645	223983	190216	794327	119670	328295	179169	85258
**Proline**	15573314	19779224	11302268	15509680	14869885	28938093	15515620	13018238	19430883	11868560	16446775	11031885	9465926
**Serine**	8394198	9359633	5098558	8786071	6591004	16096923	5747544	5918194	9636455	5338447	7864448	5263106	3347289
**Threonine**	2981382	4011665	2544511	4278168	2935498	7960906	2470637	2366324	4569895	2295261	3020304	1974069	1783652
**Tyrosine**	4540860	7318008	2346658	3027203	2725647	8794482	2048940	2176330	4829243	2100362	2888212	1999144	1520639
**Malate**	4407880	3816111	5474588	6944176	3633692	4046706	6861519	5587705	6049095	6039337	4265074	4024676	4426365
**Myristate**	1209455603	1502979552	1205885891	1252395244	1263646072	1310811211	1342953856	1753466711	1317396913	1365216179	966515910	882006076	1545704786
**Palmitate**	20211241384	19993826724	18424817346	18298039756	18974840938	20334585527	21041047132	19212720062	23151874611	21236989672	19287429436	8676747197	##########
**Succinate**	35043836	26746866	54634837	55853549	25050703	29763584	35573676	37881002	39705570	51900738	50190736	39257887	41455229
**Tryptophan**	1420859	2250390	861913	1593381	1567586	5102203	906936	1170101	2840495	872150	1360740	915670	524538
**Valine**	11726897	18875045	21953844	44422826	29481037	45675218	39096352	16776856	50662215	38708379	45150099	13247479	18074976
