# Supplementary material for: Clinical-Grade Patches as a Medium for Enrichment of Sweat-Extracellular Vesicles and Facilitating Their Metabolic Analysis
Source: Int J Mol Sci. 2023 Apr 19;24(8):7507. doi: 10.3390/ijms24087507 (PMC10139190; doi:10.3390/ijms24087507)
Supplement: Supplementary file 1 [file ijms-24-07507-s001.zip › ijms-2248755-supplementary2.pdf]

Supplementary Materials

Materials

Sorbact® superabsorbent dressing 10 x 10 cm (cat. no. 98501)

Tagaderm™ Roll 15 cm (cat. no. 16006)

40 µm sterile cell strainer (Thermo Fisher Scientific®, cat. no. 22363547)

Minisart® 0.8 µm syringe filter (Sartorius, cat. no.16592-K)

Henke-ject® 50 ml (Ref # 8300006680) Syringe

Nalgene 0.8 µm vacuum filter units (Thermo Fisher scientific)

Henke-ject® 1 ml (Ref #) Syringe

ExoView™ Human Tetraspanin Kit Ref. EV-TETRA-C. (NanoView Biosciences)

ExoView™ Human Tetraspanin Kit, Ref. EV-TETRA-C-CAR. (NanoView Biosciences)

1.5 ml Protein Lo-bind Eppendorf tube (Eppendorf®, cat. no. 022431081)

50 ml Falcon tube (Greiner, Sigma)

Nalgene® round ultracentrifuge tubes 3110-0380 38ml

Thermo Scientific™ Nalgene™ Rapid-Flow™ Sterile Single Use Vacuum Filter Units (0.8 µm)

Macroduct® Sweat Collectors

Sterile scissors, glass rod, and spatula

Spray bottle

Sterile beaker (250ml)

Sterile disposable 5, 10 and 25-ml serological pipettes for

Pipetboy (VWR)

Equipment:

Sorvall Ultracentrifuge Machine WX ultra 90 (VWR, Thermo Electron Corporation)

Sorvall AH629 rotor

ExoView™ R100 (Nanoview Biosciences Inc.)

Macroduct® advanced Model 3710 SYS (Elitech group Biomedical Systems)

Macroduct® advanced Supply Kit SS-268 (Elitech group Biomedical Systems)

Nanosight LS300

Tecnai Spirit G2 transmission electron microscope.

Chemicals

Dulbecco's Phosphate Buffered Saline without calcium and magnesium, 10X, catalog # 20-031-CV (Corning, New York, USA)

Pilocarpine hydrochloride P6503 (Sigma Aldrich)

Magnesium sulphate-7-hydrate (Riedel-de-Haen)

Standard Agarose-Type LE (BioNordika)

Antibodies

Anti-CD63 antibody (sc-5275 Santa Cruz Biotechnology, Texas, USA**Supplementary Figures**

Figure S1. ExoView analysis of sweat EVs. Image montage of the three channels, red, green, and blue showing the EVs staining with the different markers: CD63 (red), CD81 (green), and CD9 (blue).

Figure S2. CD63 expression in the sweat EVs isolated from healthy control participants. Uncropped blot of CD63 expression levels in the sweat EVs from two healthy participants (lanes 3 and 4), and negative controls (Ctrl) refer to non-enriched sweat patch, that has not been attached to a participant (lane 1) and 1x PBS (lane 2). The band of CD63 was detected at 54 kDa.

Table S1. Metabolite peak area raw data in healthy participant controls. N. number. N=11. Negative controls (N= 2) refer to the patches that were not having sweat samples.
